# Clinical analysis of 13 cases of primary squamous-cell thyroid carcinoma

**DOI:** 10.3389/fonc.2022.956289

**Published:** 2022-08-16

**Authors:** Di Ou, Chen Ni, Jincao Yao, Min Lai, Chen Chen, Yajiao Zhang, Tian Jiang, Tingting Qian, Liping Wang, Dong Xu

**Affiliations:** ^1^ Department of Ultrasonography, The Cancer Hospital of the University of Chinese Academy of Sciences (Zhejiang Cancer Hospital), Institute of Basic Medicine and Cancer (IBMC), Chinese Academy of Sciences, Hangzhou, China; ^2^ Key Laboratory of Head & Neck Cancer Translational Research of Zhejiang Province, Hangzhou, China; ^3^ The Second Clinical Medical College, Zhejiang Chinese Medical University, Hangzhou, China; ^4^ Graduate School, Wannan Medical College, Hangzhou, China; ^5^ The Postgraduate Training Base, Wen Zhou Medical University, Hangzhou, China; ^6^ Institute of Basic Medicine and Cancer, Chinese Academy of Sciences, Hangzhou, China

**Keywords:** thyroid, primary squamous-cell thyroid carcinoma, thyroid carcinoma, ultrasound, pathological features

## Abstract

**Objective:**

To analyze the clinical features, ultrasonographic manifestations, pathological features, treatment and prognosis of primary thyroid squamous cell carcinoma (PSCTC) and summarize the experience in diagnosis and treatment of this condition.

**Methods:**

A retrospective analysis was conducted on patients who were admitted to Zhejiang Cancer Hospital from 2007 to 2021 due to thyroid nodules or thyroid malignant tumors that were ultimately confirmed by postoperative pathology as primary thyroid squamous cell carcinoma. We summarize the general situation, clinical information, laboratory examination, ultrasonic image characteristics, pathological examination, clinical treatment and prognosis of the patients.

**Results:**

PSCTC is most often seen in older men and progresses rapidly. In laboratory tests, some patients had elevated levels of tumor markers (CA199, squamous cell carcinoma antigen level), thyroglobulin levels and tumor-related substances, but all these indicators lacked specificity. The ultrasound features of PSCTC are mainly hypoechoic, hard, substantial nodules with gross borders and a grade 1-2 blood flow signal, sometimes with signs of necrosis and calcification. In terms of treatment, PSCTC is mainly surgically resected, though some patients in this study underwent iodine-131 radiation therapy, local radiotherapy, and chemotherapy with unclear results. None of the patients survived for very long after treatment, but the prognosis of patients with highly differentiated squamous carcinoma was significantly better than that of patients with poorly differentiated squamous carcinoma. Papillary thyroid carcinoma may be one of the causes of PSCTC.

**Conclusion:**

PSCTC is a malignant tumor with high malignancy and rapid clinical progression. Treatment options are mainly based on surgical resection and can be supplemented with radiotherapy and chemotherapy, but there is still a lack of a standardized treatment management system, and more cases and reports are needed to accumulate data.

## 1 Introduction

Primary squamous cell thyroid carcinoma (PSCTC) is an extremely rare, aggressive and malignant tumor on the thyroid, with a very poor prognosis, accounting for about 0.1–1% of all primary thyroid malignancies (1). In the 1950s, von Karst reported this type of malignant tumor for the first time (2, 3). Although more than 60 years have now passed since then, the exact cause of this disease remains unknown. However, related studies have shown that the age of the patient at the time of onset and the tumor grade at the time of treatment are closely related to tumor size and overall survival (OS) (4). Currently, there are no specific treatment guidelines or programs for PSCTC anywhere in the world, and most patients die within one year of onset. Early detection and diagnosis are therefore essential for the treatment of PSCTC (5, 6).

The diagnosis of PSCTC relies on ultrasonography, CT and histopathology. Treatment is mainly surgical and is supplemented by radiotherapy and chemotherapy. However, most reports show that, even when surgery is combined with radiotherapy and chemotherapy, the survival rate remains poor.

The author of this study is based at a specialist oncology hospital and, following a thorough search through hospital files, 13 cases of PSCTC were found, complete with full clinical records. The close examination and analysis of these case files brings to light new insights that will improve the diagnostic and management procedures for future PSCTC patients.

## 2 Materials and methods

### 2.1 Case selection

Inclusion criteria:

1. the patient was treated in our hospital and evaluated for surgical treatment;
2. the pathological diagnosis after surgical treatment was PSCTC, which was further verified by immunohistochemistry if necessary;
3. the patient has not undergone any treatment prior to admission;
4. the patient does not suffer from other systemic malignancies.

Exclusion criteria:

1. the patient has a history of radiation exposure at an early age
2. the patient is suspected of thyroid metastasis due to other systemic malignancies;
3. the patient refuses to receive treatment.

### 2.2 Clinical characteristics

#### 2.2.1 Basic information

Patients were retrospectively analysed for age, gender, reason for consultation, family history of tumors and distant metastases.

#### 2.2.2 Laboratory examinations

Retrospective analysis of patients’ thyroid function, tumor markers, tumor-related substance levels and genetic test results.

#### 2.2.3 Ultrasound examination

Retrospective analysis of ultrasound images of squamous thyroid carcinoma. The main features summarized are size, location, echogenicity, borders, blood flow signal, elastography, and calcification of PSCTC on ultrasound images.

#### 2.2.4 Pathological

Complete post-operative pathology was available for each of the 13 patients, 11 of whom had immunohistochemistry results and, in addition, we recorded the size of each patient’s PSCTC, surrounding tissue invasion, lymph node metastases and pathology of other glandular lobes.

#### 2.2.5 Treatment and follow-up

Every patient with PSCTC was treated surgically and we recorded the type of surgery, the date of surgery, details of other treatments that some of the patients underwent (iodine-131 radiation therapy, local radiotherapy and chemotherapy), the date of recurrence, and the date of death.

## 3 Results

### 3.1 Basic Information

A total of 13 patients were included in the study, aged 52-75 years, all of whom were treated between 2007 and 2022 and all of whom had surgically resected pathology specimens. This cohort consisted of nine males and four females; 12 of the 13 patients were first-time patients; and one had a recurrent papillary thyroid carcinoma following surgery at another hospital. All patients were diagnosed with significant clinical symptoms such as palpable neck swelling, dysphagia, hoarseness and throat discomfort. Two patients had a family history and a further two had pulmonary metastases at the time of presentation (**Table 1**).

**Table 1:** Basic clinical information of 13 patients with PSCTC.

Patient No.	Gender	age	Reasons for consultation	Family history of tumors	Distant metastases
1	male	75	Dysphagia, mild dyspnea	None	None
2	male	65	Hoarse voice, choking and coughing with water	Brother with gastric cancer	None
3	male	51	Left neck mass for 7 years	None	None
4	male	58	Throat discomfort for half a month	None	None
5	male	54	Palpable walnut-sized nodule on the right side of the neck for 3 months, unbearable pins-and-needles pain on the right side of the face for 2 months, hoarse voice for 5 days	None	None
6	female	75	Left neck mass palpable for 1 month, obstruction to eating, hoarseness	None	Secondary malignant neoplasm of the lung
7	female	78	Left neck swelling increased from 1cm to 5cm in four months	None	None
8	female	60	Left neck nodule of about 2cm found 1 month	None	No metastases at presentation, metastases in lungs later in treatment
9	male	62	Hoarseness for 20 days	None	None
10	female	74	Cough with breathlessness for 2 months	None	None
11	male	61	Neck swelling for 10 years with pressure symptoms for 1 month	None	Secondary malignant neoplasm of the lung
12	male	73	After thyroid cancer resection, the recent swelling in the thyroid area continues to increase in size	None	None
13	male	52	Coughing for 1 month	Father with liver cancer Daughter with thyroid cancer	No metastases at presentation, metastases in lungs and brain late in treatment

### 3.2 Laboratory examination

Some of the patients did not receive a complete suite of laboratory tests at the time of their admission.Only seven of the patients had a full range of tumor markers at the time of their initial consultation; three of these seven patients had CA199 levels above normal and three had squamous epithelial cell carcinoma antigen levels above normal. Of note, one patient (#10) had a post-operative pathology of papillary thyroid carcinoma at the time of initial diagnosis and had a normal squamous epithelial cell carcinoma antigen level at that time, but when the neck mass recurred and the surgical pathology confirmed the diagnosis of PSCTC, the level of this tumor marker was significantly above normal. Thyroid function indicators were recorded in 12 patients, eight of whom had thyroglobulin levels above normal. Of note, four patients had tumor related substances tested, three of whom had significantly elevated genetic tumor-related material (GTM), two had elevated polysaccharide tumor related material (PTM) and enzymatic tumor-related material (ETM), and one had elevated hormonal tumor-related material(HTM). In addition, one patient had significantly elevated CK20, MMP9 and CK19 genetic tests.In addition, one patient was tested for CK20, MMP9 and CK19, and the results were positive for mutations.

### 3.3 Ultrasound

**Figure 1:**
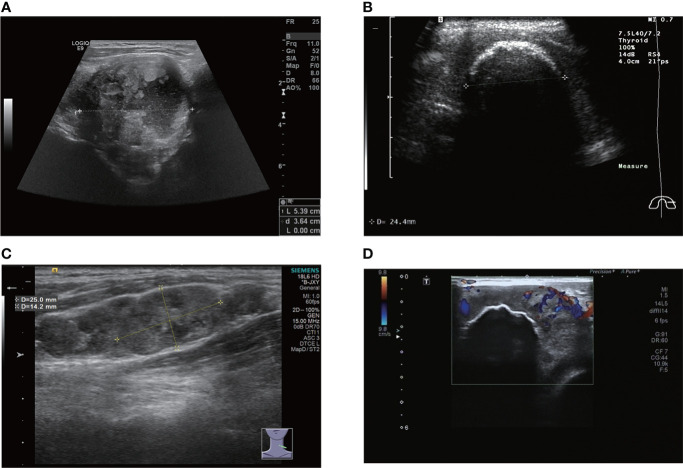
**(A)** large nodule with internal necrosis. **(B)** there is a large, eggshell-like calcification on the surface of the nodule; information on the interior is not accessible. **(C)** metastatic lymph nodes appear like beads on the left side of the neck. **(D)** there is a large calcification inside the nodule and CDFI shows a grade 2 blood flow signal.

**Figure 2:**
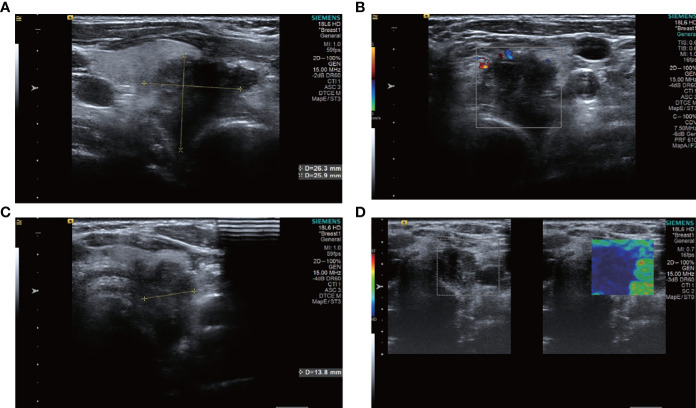
ultrasound images of patient #2, showing a hypoechoic nodule with indistinct borders in the left lobe of the thyroid gland, which was clearly visible as a nodule breaking through the thyroid peritoneum and invading the surrounding tissue. The patient underwent surgery only and had a survival period of 2 years and 7 months. **(A)** Sagittal scan **(B)** CDFI **(C)** Transverse scan **(D)** Elastography.

**Figure 3:**
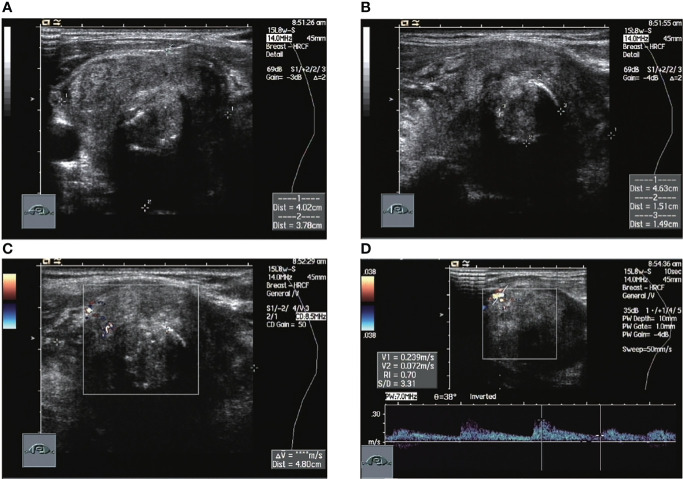
Ultrasound images of patient #5 in which normal thyroid tissue is no longer visible. The nodule occupies the entire image, with annular calcifications visible within it and a relatively rich local blood flow signal of RI=0.7. This patient had a recurrence 2 months after surgery, was treated with radiation after a second operation and was finally lost to follow-up. **(A)** Sagittal scan **(B)** Transverse scan **(C)** CDFI **(D)** Pulsed doppler.

**Figure 4:**
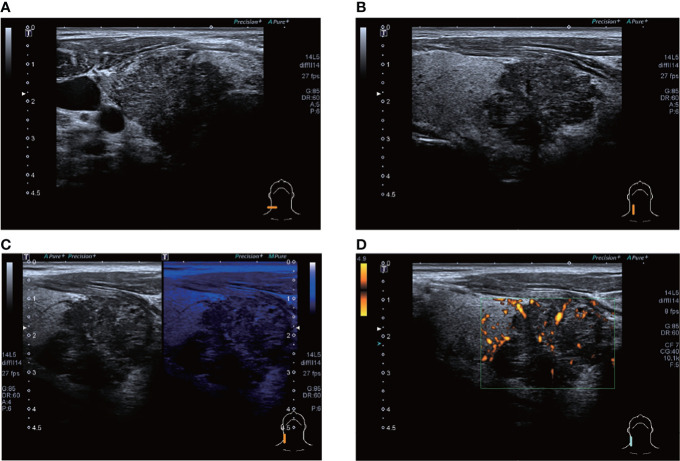
ultrasound images of patient #4 showing a hypoechoic nodule, which appears to be lobulated, in the lower pole of the right lobe of the thyroid gland, with punctate calcifications visible within. CDFI shows a grade 1 blood flow signal. The patient’s postoperative pathology was that of a highly differentiated squamous carcinoma, which has not recurred to date. **(A)** Lateral scan **(B)** Sagittal scan **(C)** "Firefly" technique **(D)** PDI.

A total of 12 of the 13 patients had complete ultrasound imaging and one patient did not have a repeat examination because he had ultrasound imaging at another hospital (**Table 2**). Eleven patients had never undergone surgery prior to admission and one was a patient who had a recurrence after surgery at an external hospital (#10). The available ultrasound images showed hypoechoic nodules in all patients except for one, who had a large, strongly echogenic spot in the lesion for which no specific ultrasound features could be obtained. Areas of necrosis due to excessive nodal growth were visible in the central area of some of the nodules (**Figures 1A, B**). All nodules were poorly defined, with some having burr-like borders and a distinct outward invasive character (**Figures 2A, C**, **3A, B**). The blood flow signal of the lesion is generally at grade 1-2 (**Figures 1D**, **2B**, **3C**, **4D**) and some of the nodules can be seen to be very hard in texture by the use of ultrasound elastography (**Figure 2D**). (Only ultrasound images of occupancies with clear PSCTC in the pathological diagnosis were recorded.)

**Table 2:** Ultrasonographic manifestations of PSCTC.

Patient No.	Size	echo	boundary	CDFI (Grade)	Ultrasound elastography	Position	Calcification
1	48mm*57mm*56mm	Low	unclear	2	4	Left lobe of the thyroid	Rod-shaped calcification
2	26mm*26mm*14mm	Low	unclear	1	5	Left lobe of the thyroid	None
3	54mm*50mm*39mm	Low	unclear	1	/	Left lobe of the thyroid	None
4	32mm*25mm*32mm	Low	unclear	2	2	right lobe of the thyroid	Punctate calcification
5	48mm*40mm*38mm	Low	unclear	2	/	right lobe of the thyroid	Cyclic calcification
6	On examination a large, curved calcification can be seen, the size of the nodule and the ultrasound situation are difficult to assess	/	/	/	/	Left lobe of the thyroid	Eggshell-like calcification
7	Nodules too large to measure	Low	unclear	2	5	Left lobe of the thyroid	Eggshell-like calcification
8	22mm*23mm	Low	unclear	1	/	Left lobe of the thyroid	None
9	21mm*22mm*29mm	Low	unclear	1	/	Left lobe thyroidectomy area	None
10	30mm*27mm*28mm	Low	unclear	1	4	right lobe of the thyroid	Rod-shaped calcification
11	34mm*28mm*21mm (left), 44mm*51mm*29mm (right)	Low	unclear	2	/	Bilateral lobes of the thyroid	None
13	37mm*36mm*33mm	Low	unclear	1	/	Right upper mediastinum	None

### 3.4 Pathology

Of the 13 patients, seven had papillary thyroid carcinoma and one had sarcomatoid carcinoma. Six patients had carcinoma in the left lobe of the thyroid, two in the right lobe, two in both the left and right lobes, one in the right side of the upper mediastinum, one postoperative recurrence in the left lobe of the thyroid in the excisional area, and one postoperative recurrence in the anterior cervical area. The two recurrent cases were both papillary thyroid carcinomas at the initial surgery and had a squamous component in the recurrent lesions. All PSCTCs had varying degrees of infiltration or metastasis to surrounding tissues. In addition, four patients had concomitant papillary thyroid carcinoma in the gland of the contralateral lobe or non-squamous carcinoma lesion.

### 3.5 Treatment and follow-up

All 13 patients were treated surgically, with seven receiving surgical treatment only. The pathology of patient #4 was highly differentiated squamous carcinoma and he was regularly followed up in 2018 after surgical treatment with no tendency to recur. Two of these patients were lost to follow-up (#1 and #7) and four patients died, with survival periods of 31 months (#2), 18 months (#6), 5 months (#10), and 15 months (#11). Four patients were treated with surgery plus iodine-131 radiation therapy/local radiotherapy/chemotherapy after recurrence. The time to recurrence in these four patients was: 1 month (#5), 4 months (#8), 17 months (#9), and 12 months (#12). Two of the four patients were lost to follow-up and the survival period for the two remaining patients was: 38 months (#9) and 62 months (#12).

One patient (#2) was lost to follow-up after treatment with simustine capsules after surgery in two separate sessions. One patient (#13) developed brain metastases 1 month after surgery in two separate sessions and passed away 6 months after chemotherapy, with an overall survival period of 8 months.

## 4 Discussion

Primary PSCTC is an extremely rare neoplasm of the thyroid, first identified by Von Karst in 1950 (2, 3), that accounts for less than 1% of all primary thyroid malignancies. In the process of the diagnosis and treatment of thyroid cancer, our most common type of cancer is papillary thyroid carcinoma (PTC) which generally has an extremely good prognosis and can lead to doctors relaxing their vigilance. There is a foreign study on the database of the American Surveillance, Epidemiology, and End Results (SEER) Program, overviewing the cases of PSCTC from 1973 to 2012 (5–7), and a total of 199 cases of PSCTC were found.

### 4.1 Clinical symptoms

This study found that 48.5% of the tumors had a maximum diameter ≥50 mm and the average tumor size was 51.5mm. The median survival period is 9 months. This is a very similar biological profile to that of anaplastic thyroid cancer. All these patients had postoperative histopathological confirmation of PSCTC and were first seen because of symptoms related to the neck, rather than as a result of a physical examination. This suggests that PSCTC, unlike most occult thyroid cancers, has a rapid clinical progression and the rapid growth of the tumor is evident to the patient. One patient had what appeared to be a walnut-sized swelling on the right side of the neck three months prior to presentation, and one month later developed facial pain, suggesting that the tumor had invaded the nerves during the course of just one month. After another month, this same patient developed a choking cough when drinking water, indicating that the laryngeal nerve was also infiltrated. Another patient had a neck mass that grew from 1cm to 4cm in diameter in four months, a rapid growth and invasion that is rarely seen in patients with differentiated thyroid cancer.

### 4.2 Auxiliary examination

The laboratory tests for PSCTC are not specific and almost every patient already has abnormal indicators, but they are not centralized. It is worth noting that squamous epithelial cell carcinoma antigen levels appear to show an upward trend with patient recurrence. One patient had a normal squamous epithelial cell carcinoma antigen level at the time of initial diagnosis but, by the time of recurrence, this was showing a tendency to increase, which may provide a new clinical indicator for postoperative review and detection of recurrence in patients with PSCTC. In addition, ultrasonography is clinically important for the detection of thyroid lesions, except in cases of PSCTC. Overall, the ultrasound features of PSCTC are hypoechoic, poorly defined, with a grade 1-2 flow signal, hard and partially associated with calcification and necrosis. PSCTC on ultrasound images has significant imaging features that differ from differentiated thyroid cancer, including severe invasion into surrounding tissues and a large mass size, which also suggests an unsatisfactory prognosis through imaging. The diagnosis of PSCTC still relies mainly on histopathology, and no comprehensive studies have been reported specifically on the preoperative examination of PSCTC. The imaging of PSCTC almost always shows signs of thyroid occupancy, compression of surrounding tissues such as displacement of the trachea and esophagus, and enlargement of multiple lymph nodes in the neck.Because PSCTC is a rare disease, genetic testing has not been carried out on a large scale. One patient was tested for CK20, MMP9 and CK19 in the blood, and the results were positive for mutations, but genetic testing is still of extremely limited diagnostic and therapeutic relevance.

### 4.3 Pathogenesis

The thyroid is a glandular tissue that secretes thyroid hormones and does not have squamous epithelial tissue, so the origin of PSCTC is not clear. Various hypotheses have been proposed, including the embryonic nest theory, postulating that squamous cells originate from remnant cells from the thyroglossal duct or thymic epithelium (8). Alternatively, the theory of dedifferentiation has been put forward, suggesting the idea that PSCTC may have been dedifferentiated from other adenocarcinomas (9). Thewjitcharoen (2020) reported a case of a 79-year-old female patient who had undergone PTC surgery and then relapsed with a diagnosis of PSCTC, validating the theory of dedifferentiation described above (10).

In the current study, there are similar cases (#9 and #12), both of whom had pathological findings of PTC at their initial surgery but had postoperative pathology of PSCTC at their later recurrence and re-surgery. It has also been suggested that the development of squamous thyroid cancer is related to the environment within the thyroid gland, such as autoimmune inflammation or chronic disease (11). In our study, however, the postoperative pathology did not show an inflammatory cell infiltrate and the laboratory tests did not show elevated levels of autoimmune markers.

### 4.4 Diagnosis

The diagnosis of PSCTC relies on several aspects of the development of the disease, laboratory tests, imaging and pathological diagnosis. However, the benign or malignant nature of the disease can almost always be determined in the preoperative ancillary tests, so patients are commonly treated surgically, making preoperative pathological diagnosis almost irrelevant. It is worth noting that the current study identified approximately 200 cases of squamous thyroid cancer at the time of initial screening, but after careful checking, we found that most of the squamous thyroid cancers were secondary. If a patient has a squamous component found in the goiter without any symptoms and without metastases in the neck lymph nodes, clinicians often need to review gastroscopy, chest CT, etc. Almost all secondary squamous thyroid cancers originate from oesophageal malignancies. This can be identified by immunohistochemistry. All cases in this study therefore underwent relevant ancillary investigations to exclude the possibility of metastases to the thyroid from other malignancies.

The disease history is mainly characterised by a cervical occupancy and associated compression symptoms. In laboratory tests, CA199 was elevated in three patients, GTM was significantly elevated in three patients, sugar tumor related material (PTM) and ETM was elevated in two patients, and HTM was elevated in one patient. This does not appear to be sufficiently specific and more case reports are required to verify the significance of these indicators. The level of squamous epithelial cell carcinoma antigen also appears to be significant in terms of patient recurrence.

Finally, eight of the 12 patients had higher than normal levels of thyroglobulin, and a related study reports that this probably represents non-specific absorption from adjacent cells (12).In terms of imaging, we performed mainly US and CT examinations; where the CT images predominantly showed a hypodense occupancy of the neck and associated compression symptoms, with no specific presentation, the US presentation was more specific, so we performed a detailed analysis of each patient. The signal was hard, with calcification and necrosis visible in some parts (**Figures 1**-**4**).

### 4.5 Pathology

**Figure 5:**
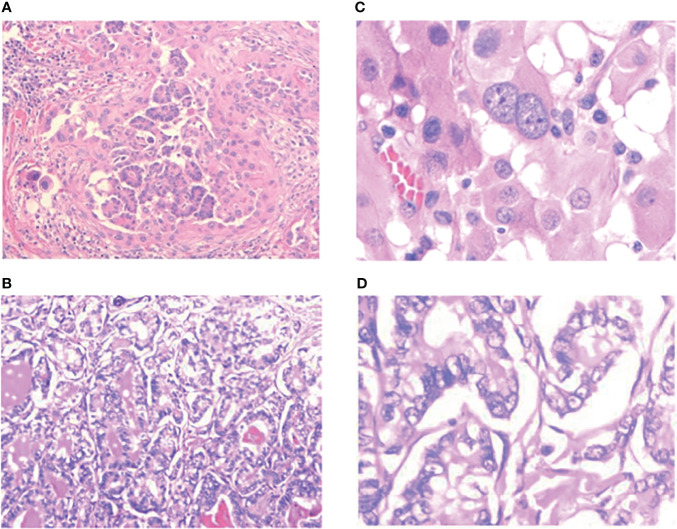
Pathological images of PSCTC. **(A, B)** 100×, **(C, D)** 400×.

**Figure 6:**
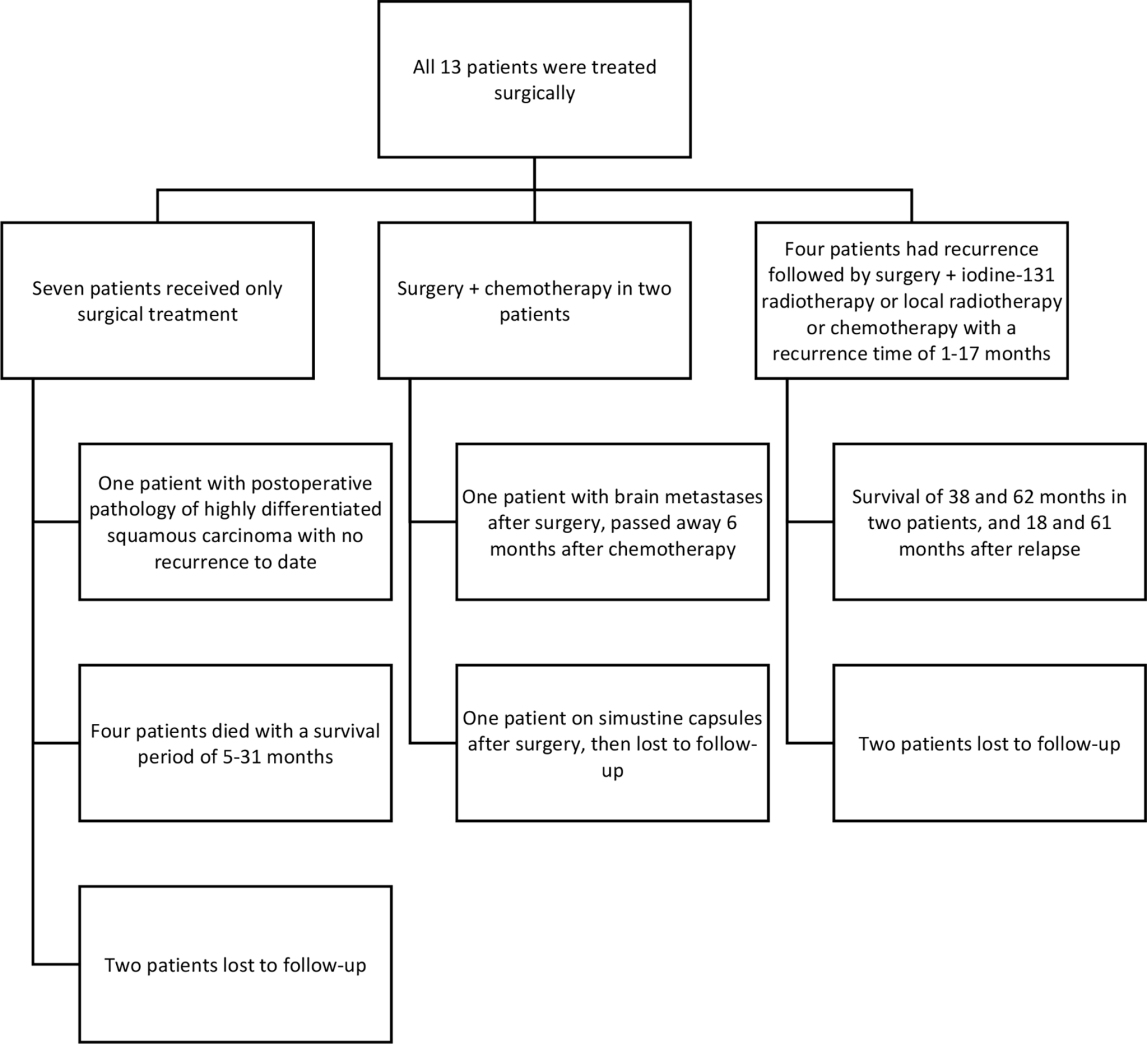
Treatment and outcomes for patients with PSCTC.

In the process of summarizing the postoperative pathology of the 13 PSCTC cases, we made some findings that are shown in **Figures 5**, **6**, and in **Table 3**.

**Table 3:** Treatment and follow-up of PSCTC.

patient	time of first operation	Initial surgical approach	Recurrence time	Re-operation time	Re-operative approach	Other treatments	Ending
1	2019-11-26	Total thyroidectomy + bilateral central zone lymph node dissection + bilateral cervical lymph node dissection	lost to follow-up				
2	2018-5-25	Left lobe thyroidectomy + bilateral central lymph node dissection					2020-12-5 death
3	2007-5-21	Left lobe thyroidectomy + lymph node dissection of the left central region + lymph node dissection of the left lateral neck	2007-8-30	2007-7-3	Major right lobe thyroidectomy + right central lymph node dissection + right lateral cervical lymph node dissection	Simustine Capsules 200mg po once	lost to follow-up
4	2018-2-23	Total thyroidectomy + right central lymph node dissection	\	\	\	\	No recurrence
5	2009-4-6	Total thyroidectomy+bilateral lateral cervical lymph node dissection	2009-5-5	2009-7-10	Lymph node dissection in the right upper cervical region	Radiation therapy	lost to follow-up
6	2007-5-25	Left lobe thyroidectomy					2008-11-15 death
7	2018-7-19	Left thyroid lobe and isthmus resection + left cervical lymph node dissection	lost to follow-up				
8	2012-9-14	Total thyroidectomy+left cervical lymph node dissection + central lymph node dissection	2013-1			2013-1-29 and 2013-7-8 Oral 131-sodium iodide solution	lost to follow-up
9	2016-10-19	Total thyroidectomy+left cervical lymph node dissection	2018-3-22	2018-4-11	Lymph node dissection of the neck	131-iodine treatment on 2017-1-6, radiotherapy started on 2018-05-08	2019-12-15 death
10	2019-3-1	Total thyroidectomy					2019-8-1 death
11	2015-8-10	Total thyroidectomy+bilateral lateral cervical lymph node dissection+mediastinal lymph node dissection					2016-11-28 death
12	2013-10-17	Partial thyroidectomy of the left lobe of the thyroid + thyroidectomy of the right lobe of the thyroid	2013-11-26	2019-4-15	Total thyroidectomy + bilateral cervical lymph node dissection	Anrotinib 12mg po qd	2019-12-26 death
13	2016-10-10	Right lobe thyroidectomy + right cervical lymph node dissection	2017-1-10	2016-12-5	Left lobe thyroidectomy + lymph node dissection of the left side of the neck	Patient presented with brain metastases in Jan 17, chemotherapy from 2017-01-10 to 2017-2-28	2017-6-7 death

Firstly, in a rather unique case, patient #4 had a highly - moderately differentiated squamous cell carcinoma; she was only surgically resected in 2018, but the patient has been recurrence free remains clinically cured to date. This shows that not every PSCTC has a high recurrence rate and high mortality as perceived, and that PSCTC should also be treated aggressively in clinical practice. In addition, we found that PSCTC appears to be closely related to PTC, with postoperative pathology in patients #1, #3, #8 and #13 all showing that PTC was found in the lobe of the gland without PSCTC. Postoperative pathology in patients #1, #3, #5, #6, #8, #12 and #13 all showed that PSCTC was found in PTC, and therefore may be related to the origin of PSCTC. Patient #12 had a thyroidectomy and was diagnosed with PTC on postoperative pathology, but the pathology of recurrence 5 years later was suggestive of PSCTC, which is also very intriguing (**Table 4**).

**Table 4:** Pathological characteristics of patients with PSCTC.

Patient No.	Size (cm)	location	Pathological	Invasion of surrounding tissues	Number of lymph node metastases	Immunohistochemistry	Other
1	7.5*7.4	Left lobe of the thyroid	Squamous cell carcinoma and papillary carcinoma	none	21	CD117(-), CD5(-), CK5/6(+), Ki-67(+,70%), P40(+), P53(+,70%), P63(+), PAX8(Weakly+), CK7(+), EBER(-), TG(-), TTF-1(+)。	Double papillary carcinoma of the right lobe of the thyroid
2	3*2.5*1.5	Left lobe of the thyroid	Intermediate-to-low differentiated squamous cell carcinoma	Invasion of nerves, pre-tracheal fibers, adipose tissue	1	CK(+), CD5(Lymphocytes+), CD117\c-kit(-), CHG-A\CgA(-), Sy(-), P40(+)。	
3	5.4*5.2	Left lobe and isthmus of the thyroid	Squamous cell carcinoma and papillary carcinoma	Involvement of peri-thyroidal tissue, left neck, transverse muscle tissue	23	LCA (-), CK(+++), EMA(-), TG (-), TTF1(+)	Papillary carcinoma of the right lobe of the thyroid
4	2.5cm	right lobe of the thyroid	Highly- to moderately-differentiated squamous cell carcinoma	Involvement of the peritoneum, metastasis, infiltration of thymic tissue	0	CK19(+), EMA(-), CD117\c-kit(-), CD5(-), CD56(-), Sy(-), CHG-A\CgA(-)。	
5	2.5*2.5*2cm	right lobe of the thyroid	Papillary thyroid carcinoma, poorly differentiated in most areas, with some areas with squamous differentiation	involvement of nerves	4	none	This patient had a recurrence of metastatic squamous cell carcinoma in the posterior right neck
6	5cm*6cm*5cm	Left lobe of the thyroid	Papillary thyroid carcinoma with squamous cell carcinoma in part	Infiltrating the outer lining of the esophageal wall		TG (+), TTF1(+), P53 (-), bcl-2 (-), CT (-), CK(+++), SP-A(-)	
7	10*6.4*4.5cm	Left lobe of the thyroid	Squamous cell carcinoma, sarcomatoid carcinoma in most areas	Choroidal aneurysm embolus involving the perineum, (left neck) dermis and subcutaneous fibrous and adipose tissue of the thyroid gland	2	CK(partial +), EMA(partial +), Vim(partial +), CD5(-), CD117\c-kit(-), TTF1(+), TG(灶+), PAX-8(+), Sy(-), CHG-A\CgA(-), CD56(+), CT(-), bcl-2(partial +), CD34(-), P40(partial +), Ki-67(+,Hotspots70%)。	
8	2.5×2×2cm	Left lobe of the thyroid	Papillary thyroid carcinoma, partly squamous cell carcinoma, with poorly differentiated focal areas and sarcomatoid carcinoma changes	Carcinoma infiltrating or metastasizing to fibrous, fatty, transverse muscle tissue (left cervical region III)	5	CD5(-), CD117\c-kit(-), CK19(+), TG(partial +), TTF1(+), Vim(+), CK(+)。	Papillary carcinoma of the right lobe of the thyroid
9	2.8*2.5*1.7	Left lobe thyroidectomy area	Primary squamous carcinoma of the thyroid (from squamous carcinomatosis)	Infiltration of squamous cell carcinoma in fibrous tissue (left thyroid area)	0	none	
10	Tumour in the right lobe and isthmus of the thyroid gland 5 x 4 x 3 cm, tumour in the left lobe of the thyroid gland 4 x 4 x 3 cm	The right lobe, isthmus and left lobe of the thyroid	Undifferentiated carcinoma with squamous differentiation	Involvement of peritoneal fibrous tissue, extraperitoneal transverse muscle tissue. (tracheal mass) hypofibrotic carcinoma (consistent with metastatic thyroid cancer, 1.6 x 1 x 1 cm) in submucosal fibrous tissue, involving tracheal cartilage	0	CK(+), EMA(partial +), Vim(partial +), TG(-), TTF1(-), CT(-), P40(+), P63(+), CK19(-), Gal-3\Galectin-3(+), Ki-67(+,30%)。	
11	Maximum left tumor diameter 2.2cm, maximum right tumor diameter 3.0cm	Bilateral lobes of the thyroid	Undifferentiated carcinoma with squamous differentiation	Nerve involvement, metastasis to fibrous tissue (left venous angle) with carcinoma nodule formation	1	CK5/6(+), P40(+), TG(-), Gal-3\Galectin-3(partial +), CD56(-), CD5(-), CD117\c-kit(-), TTF1(-)。	
12	6×5×4cm	Secondary (anterior neck swelling)	Papillary thyroid carcinoma with most areas of moderate-to-lowly differentiated squamous carcinoma	Extensive involvement of peripheral fibers, transverse muscle, cartilaginous tissue, involvement of the larynx and upper trachea	14	TTF1(partial +), TG(partial +), P40(partial +), P63(partial +), CK5/6(partial +), HBME-1(+), Gal-3\Galectin-3(partial +), CK7(+), P16(partial +)。	
13	4×3×2cm	Right upper mediastinum	Papillary thyroid carcinoma with intermediate to poorly differentiated squamous cell carcinoma	Involvement of the thyroid tegument, nerves	15	TG(-), TTF1(-), PAX-8(+), CK(+), EMA(+), Vim(+), P63(+), 34βE12(+), P40(+), CK5/6(+), S-100(+), Ki-67(+,40%), P53(+), CD5(-), CD117\c-kit(-)。	Papillary carcinoma of the bilateral lobes of the thyroid

### 4.6 Treatment and Survival

In terms of survival, all patients were either lost to follow-up or died, with the singular exception of #4, mentioned above, who remains clinically cured to this day. In previous articles, the prognosis for patients diagnosed with PSCTC, whether treated or untreated, has been poor (13).

In examining the history for the patients in this study, it is clear that most doctors recommended a post-operative combination of radiotherapy and/or chemotherapy; however, some patients abandoned further treatment because of their advanced age and frailty. Patients who received only surgery had a survival of 5-31 months and those who received radiotherapy and chemotherapy had a survival of 9-62 months. Although the sample is small, the current data shows that patients who received combined radiotherapy and chemotherapy after surgery did have better outcomes than those who received only surgery.

**Figure 7:**
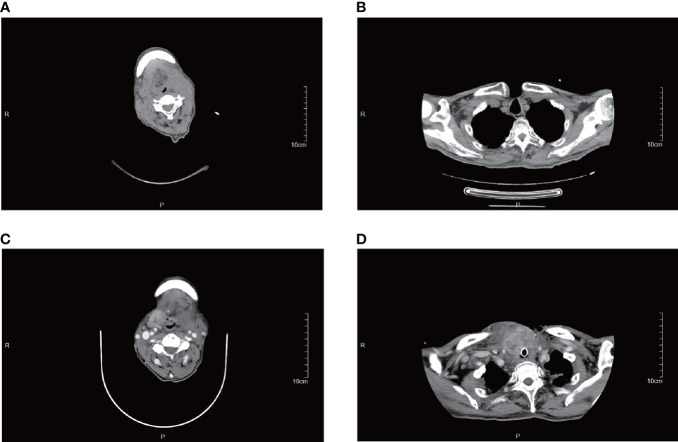
CT images of patient #12. **(A, B)** are from August 2019, and **(C, D)** are from December 2019. This patient suffered a rapid enlargement of the right upper cervical and mediastinal lymph nodes in just 4 months and died in December 2019.

Some patients were treated with surgical resection followed by radiotherapy only, even though some studies indicate that PSCTC is insensitive to radiotherapy (14, 15, 16, 17). In this study, a significant reduction in the right lower cervical lymph node can be seen in patient #5, 1 month after the completion of radiotherapy to the cervical lymph nodes. Patient #12, after relapse, was given oral Apatinib tablets (Ertan, 0.25g), for 3 weeks, after which he reported a significant reduction in the anterior cervical swelling, but later stopped taking the drug due to hemoptysis caused by the drug, This patient eventually died of a recurrence of the tumor (**Figure 7**). All of these indicate that radiotherapy and chemotherapy in addition to surgery can inhibit the growth of the tumor to some extent.

## 5 Conclusion

PSCTC is a very rare form of thyroid cancer. The diagnosis requires adequate evaluation, which can be further assessed in terms of laboratory tests and molecular examinations. Current treatment is mainly surgical, followed by a combination of radiotherapy and chemotherapy, but the prognosis for patients is generally poor. Our research center is a provincial cancer hospital but, despite this, there have only been 13 presented since 2007, which gives us an inescapably small sample. Successful clinical management of PSCTC requires a large number of case reports from which a greater amount of data and experience can be extracted.

## Data availability statement

The original contributions presented in the study are included in the article/supplementary material. Further inquiries can be directed to the corresponding authors.

## Ethics statement

The studies involving human participants were reviewed and approved by Medical Ethics Committee of Zhejiang Cancer Hospital. Written informed consent for participation was not required for this study in accordance with the national legislation and the institutional requirements.

## Author contributions

DO proposed the idea and wrote the main article, CN wrote and revised the article, JY, ML, CC, YZ, TJ, TQ collected and analysed the data, LW and DX guided the article and provided financial support. All authors contributed to the article and approved the submitted version.

## Funding

National Natural Science Foundation of China (82071946)Zhejiang Provincial Natural Science Foundation (LZY21F030001) Zhejiang Provincial Medical and Health Science and Technology Program Project (2021KY099) Zhejiang Province Science and Technology Plan of Traditional Chinese Medicine (NO.2020ZB033) Zhejiang Provincial Natural Science Foundation of China (LY20H180001).

## Conflict of interest

The authors declare that the research was conducted in the absence of any commercial or financial relationships that could be construed as a potential conflict of interest.

## Publisher’s note

All claims expressed in this article are solely those of the authors and do not necessarily represent those of their affiliated organizations, or those of the publisher, the editors and the reviewers. Any product that may be evaluated in this article, or claim that may be made by its manufacturer, is not guaranteed or endorsed by the publisher.
